# Prevalence and Clustering of Cardiovascular Disease Risk Factors among Adults Along the Lancang-Mekong River: A Cross-Sectional Study from Low- and Middle-Income Countries

**DOI:** 10.5334/gh.1319

**Published:** 2024-04-17

**Authors:** Min Ma, Liping He, Huadan Wang, Mingjing Tang, Da Zhu, Labee Sikanha, Sokha Darapiseth, Jiang Lu, Yu Xia, Zhongjie Wang, Xia Wu, Qiuyan Zhu, Lin Duo, Xiangbin Pan, Linhong Pang

**Affiliations:** 1 Affiliated Cardiovascular Hospital of Kunming Medical University, Fuwai Yunnan Cardiovascular Hospital, Kunming, China; 2 Kunming Medical University, Kunming, China; 3 Saimangkorm International Hospital, Oudomxay province, Lao PDR; 4 Referal Hospital, Ratanakiri province, Cambodia; 5 Yunnan Center for Disease Control and Prevention, Kunming, China; 6 Department of Structure Heart Center, Fuwai Hospital, National Center for Cardiovascular Diseases, Chinese Academy of Medical Sciences and Peking Union Medical College, Beijing, China

**Keywords:** Cardiovascular disease risk factors, Prevention, Low and middle-income countries

## Abstract

**Background:**

Progress in cardiovascular health is increasingly concentrated in high-income countries, while the burden of cardiovascular disease (CVD) is high in low- and middle-income countries, a clear health inequity that must be urgently addressed.

**Objective:**

This study aims to evaluate the prevalence and clustering of CVD risk factors in the three Lancang-Mekong regions.

**Methods:**

We conducted a population-based cross-sectional survey from January 2021 to March 2023 in China, Laos, and Cambodia. We compared the prevalence and clustering of CVD risk factors–including hypertension, dyslipidemia, diabetes mellitus, overweight/obesity, current smoking status, current drinking status, inadequate vegetable and fruit intake, and insufficient physical activity–across the three regions, further stratifying the data by gender and age. Multivariate logistic regression models were performed to explore factors influencing the aggregation of CVD risk factors (≥2, ≥3, ≥4).

**Results:**

A total of 11,005 adults were included in the study. Hypertension emerged as the primary metabolic risk factor in Laos (36.8%) and Cambodia (23.5%), whereas overweight/obesity was the primary risk factor in China (37.6%). In terms of behavioral risk factors, participants in all three regions showed insufficient vegetable and fruit intake. The prevalence of individuals without CVD risk factors was 10% in China, 1.9% in Laos, and 5.2% in Cambodia. Meanwhile, the prevalence of two or more risk factors was 64.6% in China, 79.2% in Laos, and 76.0% in Cambodia. Multivariate logistic regression models revealed that the propensity for CVD risk factors clustering was higher in men and increased with age in all three countries.

**Conclusions:**

CVD risk factors and multiple clustering are pressing health threats among adults in low- and middle-income areas along the Lancang-Mekong River Basin. This study highlights the urgent need for proactive tailored strategies to control CVD risk factors.

## Introduction

Cardiovascular disease (CVD) is the major cause of death worldwide, constituting 32% of all recorded deaths [1,2]. Previous studies have reported that more than 80% of these deaths were in low- and middle-income countries (LMICs), with 58% accounting for Asia [3,4]. In 2019, CVD-related deaths in China, Laos, and Cambodia contributed to 43.04%, 27.36%, and 30.45% of total national mortalities, respectively [5]. Reducing the preventable mortality of CVD is currently one of the most important priorities in global health [6]. Up to 80% of premature heart attacks and strokes are preventable [7], and intervention with modifiable CVD risk factors is critical.

Numerous CVD risk factors, including diabetes [3,4], overweight/obesity [4], hypertension [8], tobacco use [4], insufficient vegetable and fruit intake [4,9], dyslipidemia [10], insufficient physical activity [11], and alcohol consumption [3], feature as primary, modifiable contributors to CVD. These factors have been causally linked to the emergence of acute myocardial infarction, stroke, heart failure, and cardiovascular death [12]. In a representative survey of China, 70.3%, 40.3%, and 16.7% of adults had ≥1, ≥2, or ≥3 CVD risk factors, respectively [13]. Similarly, a 2008 survey in Vientiane, the capital of Laos, found that 59.8% of the population possessed one to two risk factors, while 9.2% displayed three or more [14]. Comparable studies in Malaysia [15] and Nepal [16] revealed a heightened prevalence of aggregated CVD risk factors among adults. A previous study also compared the prevalence of hypertension, diabetes, hyperlipidemia, and behavioral risk factors control in China, Japan, and South Korea [17]. Some scholars employed a decade-spanning cross-sectional dataset to compare the metabolic and behavioral risk factors of CVD in ten Southeast Asian countries [18].

However, the reliability of using existing reports for public health guidance were often by outdated data and limited sample sizes [15,16], while multinational comparative studies were often limited by disparate survey methodologies [18], inconsistent diagnostic criteria [17], or extended data collection periods [18]. There was nearly no comparative study of CVD risk factors among adults of neighboring countries in the same region. The Lancang-Mekong River is a major transboundary river that originates in the Qinghai-Tibet Plateau in China and flows through six low- and middle-income countries (China, Myanmar, Laos, Thailand, Cambodia, and Vietnam), where about 40% of the region’s people live in poverty [48]. This study conducted cross-country population-based surveys in three provinces of the Lancang-Mekong River Basin with standardized survey procedures and the same variable definitions. The aim of this study was to describe the distribution of CVD risk factors in these low- and middle-income areas, and to assess the clustering and regional differences in risk factors.

## Methods

### Study design, settings, and population

A cross-sectional survey was conducted in three related provinces along the Lancang-Mekong River: the Yunnan province of China, the Oudomxay province of Laos, and the Ratanakiri province of Cambodia, between 2021 and 2023 (Supplementary Figure S1).

This cross-sectional study was approved by the Ethics Committee of the National Cardiovascular Center, Beijing China (No 2020-1360); Saimangkorn International Hospital, Oudomxay province Laos (No 00504); Referal Hospital, Ratanakiri province, Cambodia (No 0023). All survey participants in the study have been given the informed consent and signed consent form.

The Yunnan province is located in the southwest of China, on the upper Lancang-Mekong River, bordering Laos, with a total population of 47 million inhabitants. Detailed sampling procedures in the Yunnan province have been described in previous studies [19]. In brief, a multi-stage stratified random sampling method that selected residents aged 18 years and older who had lived locally for 6 months or older was selected between January and December 2021. A total of 8,859 samples were included in the Yunnan Province.

The Oudomxay province is located in northern Laos, the middle reaches of the Mekong River, bordering the Yunnan province of China. The total population of the Oudomxay province is about 322,000, and the per-capita GDP was about USD 1,368. A cluster sampling method was selected during the survey from September 2022 to March 2023. First, all villages in the province were divided into urban and rural areas as primary sampling units. Subsequently, two villages were selected at the urban and rural levels respectively and all eligible residents in the village were included in the survey. A total of 1,039 eligible participants were included. Ratanakiri is a province of northeast Cambodia, the lower Mekong River, as one of the lowest economic levels in the country. The province borders Laos and has a population of about 150,000 inhabitants. The population is mainly engaged in subsistence mobile agriculture, with most ethnic groups living from slash-and-burn farming and rice cultivation [20]. Its population density is 13.9 people per square kilometer, with most people living in small and separated villages. During September and December 2022, the cluster sampling method was used to select the Bar Kaev district with a relatively concentrated population in the Ratanakiri province as the survey site, two communities were selected using a simple random sampling method, and all of the selected residents were included in the survey. In this province, there were 1,107 eligible samples.

### Data collection

This survey from three provinces followed the WHO STEPS [16] method: one-to-one questionnaire interview (step 1); physical examination and blood pressure measurement (step 2); and biochemical index test (step 3).

In the Yunnan province, China, the field survey employed the structured questionnaire scheme from the National Cardiovascular Center (Beijing, China), and data collection was facilitated using iPad devices. A proficient team of 20 individuals, comprising seasoned clinicians, nurses, pharmacists, and medical students, served as investigators. They conducted face-to-face interviews with study participants, gathering information such as gender, age, place of residence, ethnicity, education level, current smoking and alcohol consumption habits, dietary intake, physical activity, and more. Following this, the investigators measured the participants’ height, weight, and blood pressure using standardized and calibrated instruments. Finally, venous blood samples were drawn from the survey participants after a fasting period of at least 12 hours, enabling tests for fasting blood glucose (FBG) and total cholesterol (TC).

For the Oudomxay and Ratanakiri provinces, investigators received comprehensive training from China, supplemented by a field investigation training video. Translated versions of the survey protocol and the questionnaire were then employed in both Laos and Cambodian languages. A local investigation team of 10–15 experienced doctors and nurses carried out face-to-face interviews with the respondents. The survey procedures, questionnaire content, protocols for physical examination, and biochemical measurements were harmonized with those applied in Yunnan province, China.

### Definition of study variables

Demographic variables included sex (man, woman), age groups (18–34, 35–49, 55–64, ≥65), ethnicity (main ethnic group and ethnic minority groups), and education level (no education, primary school, junior high school and above). Eight outcome variables were modifiable CVD risk factors according to the European Society of Cardiology [21] and the American Society of Cardiology [3] as current smoker, current alcohol drinker, insufficient physical activity, insufficient vegetable and fruit intake, overweight/obesity, hypertension, hypercholesterolemia, and diabetes mellitus (DM). Being a current smoker was defined as smoking at least 7 cigarettes per week, for at least 6 months [12]. Drinking within the past month was defined as being a current drinker [18]. According to the WHO recommendations, insufficient physical activity was defined as performing <150 min of moderate-intensity or <75 min of vigorous-intensity physical activity per week, or any equivalent combination of the two [22]. For vegetable and fruit intake, a frequency of less than 1–3 days per month was considered insufficient [23]. Body mass index [BMI] was obtained by dividing body weight by the square of the individual’s height. Based on WHO cut-off thresholds, a BMI of 25–29.9 kg/m^2^ was defined as overweight, and a BMI of 30 kg/m^2^ was defined as obese [24]. Hypertensive patients were defined as having an SBP ≥140 mm Hg and/or a mean DBP ≥90 mm Hg, or self-reporting that they had taken antihypertensive medication within the past two weeks [25]. Hypercholesterolemia was defined as serum total cholesterol ≥5.17 mmol/L [200 mg/dL] or the use of lipid-lowering drugs [26]. DM was defined as having a fasting blood glucose ≥7.0 mmol/L or taking any antidiabetic medication [27]. The clustering of CVD risk factors was assessed by the presence of eight major risk factors in an individual. The clustering of CVD risk factors was defined as the presence of any two or more risk factors [28].

### Statistical analyses

Overall participant characteristics were described by means and standard deviations or frequencies (percentages). The Chi-square test and one-way analysis of variance were used to compare the demographic characteristics and measurement index differences among the three countries. Later, the distribution of eight CVD major risk factors in the three Lancang-Mekong countries was described, and the prevalence of CVD risk factors was visualized by lollipop charts. Previous studies [17,29] have revealed sex differences in the prevalence and clustering of CVD risk factors, so we further presented a sex-stratified perspective on CVD risk factors and clusters in visual lollipop charts. Later, the overall distribution, aggregation, and crossover of CVD metabolic risk factors (hypertension, DM, hypercholesterolemia, overweight/obesity) and behavioral risk factors (current smoker, current drinker, insufficient vegetable and fruit intake, insufficient physical activity) were presented for all samples in a Venn diagram.

Furthermore, the number of CVD risk factors aggregation was presented as count variables between 0 and 8. Multivariable logistic regression models were performed for each of the three country samples, with dependent variables stratified by (≥2, ≥3, ≥4), and adjusted OR and 95% confidence interval (*CI*) were estimated. All statistical analyses were performed using the IBM SPSS 26.0 statistical software and the R 4.0.5 software (https://www.r-project.org/). Two-tailed *P* < 0.05 was considered statistically significant. If key variables for determining CVD risk factors were missing, we excluded the missing values from the relevant calculations and analyses [18]. Data analysis was conducted from April 2023 to August 2023.

## Results

### Baseline characteristics of the study population in China, Laos, and Cambodia

Table 1 showed the study included 11,005 participants aged 18 years and above across three countries. Among these, 80.5% of the participants were from Yunnan province, China (49.5% were men, mean age 44.3 ± 18.0 years), 9.4% from Oudomxay province, Laos (33.8% were men, mean age 47.2 ± 16.5 years), and 10.1% from Ratanakiri province, Cambodia (36.9%were men, aged 45.1 ± 12.4 years). While the main ethnicity of each country predominated, Laos stood out with 91.1% of subjects belonging to minority ethnic groups.

**Table 1:** Demographic characteristics of the sample population from China, Laos, and Cambodia along the Lancang-Mekong River (N, %).


	PARTICIPANTS, N. (%)				

	TOTAL	CHINA	LAOS	CAMBODIA	*P* VALUE

**Total**	11005	8859 (80.5)	1039 (9.4)	1107 (10.1)	NA

**Sex**					

**Women**	5863 (53.3)	4476 (50.5)	688 (66.2)	699 (63.1)	<0.001

**Men**	5142 (46.7)	4383 (49.5)	351 (33.8)	408 (36.9)	

**Age group, y**					

**18–34**	3610 (32.8)	3094 (34.9)	290 (27.9)	226 (20.4)	<0.001

**35–44**	2309 (20.9)	1738 (19.6)	189 (18.2)	382 (34.5)	

**45–54**	1965 (17.8)	1532 (17.3)	206 (19.8)	227 (20.5)	

**55–64**	1366 (12.4)	1025 (11.6)	170 (16.4)	171 (15.4)	

**≥65**	1755 (15.9)	1470 (16.6)	184 (17.7)	101 (9.1)	

**Ethnic group**					

**Main ethnic group**	6823 (61.9)	6190 (69.9)	92 (8.9)	541 (48.9)	<0.001

**Ethnic group**	4182 (38.0)	2669 (30.1)	947 (91.1)	566 (51.1)	

**Education**					

**No education**	1770 (16.0)	954 (10.8)	337 (32.4)	479 (43.3)	<0.001

**Primary school**	2742 (24.9)	1947 (22.0)	406 (39.1)	389 (35.1)	

**Junior high school and above**	6493 (59.0)	5958 (67.3)	296 (28.5)	239 (21.6)	

**Blood pressure, mean (SD), mm Hg**					

**SBP**	127 (20)	127 (20)	129 (25)	122 (16)	<0.001

**DBP**	80 (12)	80 (11)	81 (14)	78 (10)	<0.001

**TC, mean (SD), mmol/L**	4.59 (1.24)	4.68 (1.20)	4.00 (1.52)	4.44 (1.04)	<0.001

**FBG, mean (SD), mmol/L**	5.43 (1.89)	5.16 (1.52)	7.04 (3.23)	6.04 (1.91)	<0.001

**BMI, mean (SD), kg/m** ^2^	23.53 (3.84)	23.89 (3.83)	22.36 (3.94)	21.91 (3.08)	<0.001


Abbreviation: SBP: Systolic blood pressure; DBP: Diastolic blood pressure.

### Prevalence of CVD risk factors

**Figure 1:**
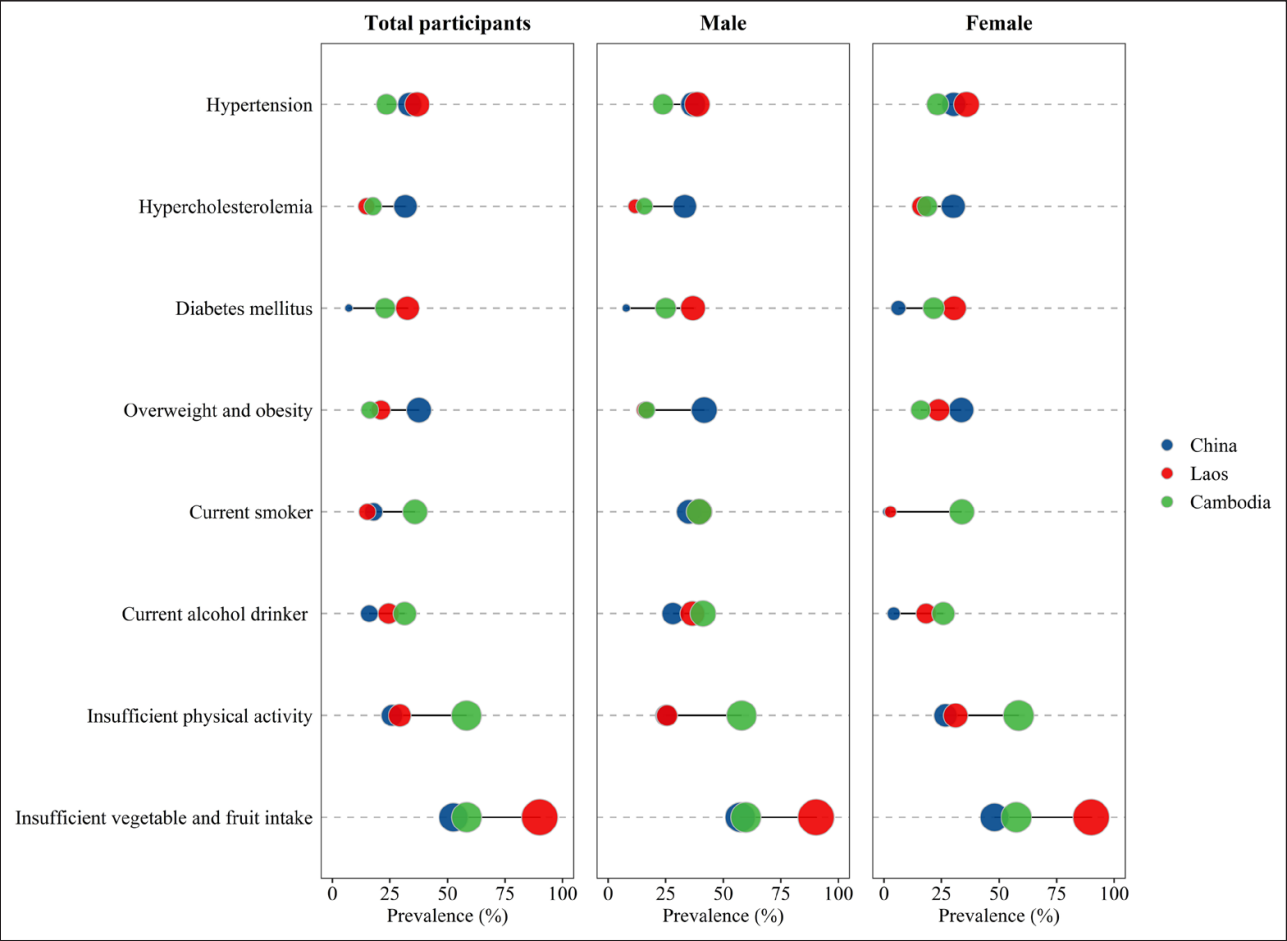
Prevalence of cardiovascular disease risk factors in the three countries.

In terms of behavioral risk factors, Laos had the highest prevalence of inadequate intake of vegetables and fruits at 90.1%, which was significantly higher than in China (52.6%) and Cambodia (58.4%). The prevalence of hypertension was highest in Laos (36.8%), followed by Cambodia (32.6%). Furthermore, over half of Cambodia’s participants (58.3%) reported insufficient physical activity, while smoking (35.9%) and current drinker (31.4%) rates were elevated in comparison to the other two countries. On the other hand, registered the highest rates of overweight/obesity (37.6%) and dyslipidemia (36.8%). With the exception of physical inactivity, which was more pronounced in women, and dyslipidemia, which was more prevalent in women from Laos and Cambodia, other prevalence rates and risk factors were higher among men than women (Figure 1).

### Metabolic and behavioral risk factors in the participants

**Figure 2:**
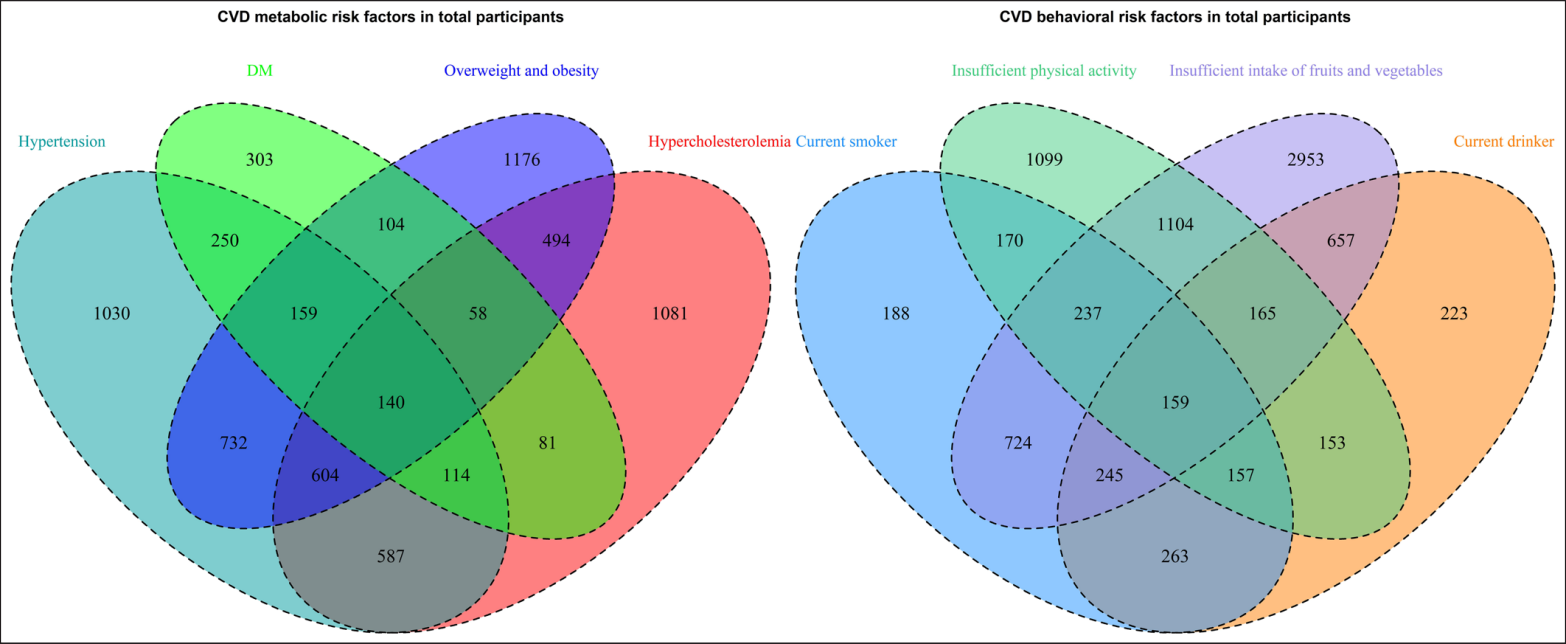
Cluster distribution of cardiovascular disease risk factors from the total sample of the three countries.

For the CVD metabolic risk factors, hypertension took the lead with the highest count (n = 3616). Among the combinations of two metabolic risk factors, hypertension and overweight/obesity coexisted most (n = 1635). Of the three risk factor combinations, the cluster consisting of hypertension, DM, and overweight/obesity had the highest prevalence (n = 744). Shifting focus to CVD behavioral risk factors, the most frequently encountered factor was insufficient intake of vegetables and fruits, totaling 6,244. Inadequate intake of vegetables and fruit alongside insufficient physical activity had the highest number of dual behavioral risk factor combinations (n = 1635). In addition, the combinations of smoking, being a current drinker, and having an inadequate vegetable and fruit intake were the most common of the three risk factors clustering (Figure 2). A detailed breakdown of the clusters of metabolic and behavioral risk factors in each of the three regions can be found in Supplementary Table S1 and Table S2.

### Clustering of CVD risk factors

**Figure 3:**
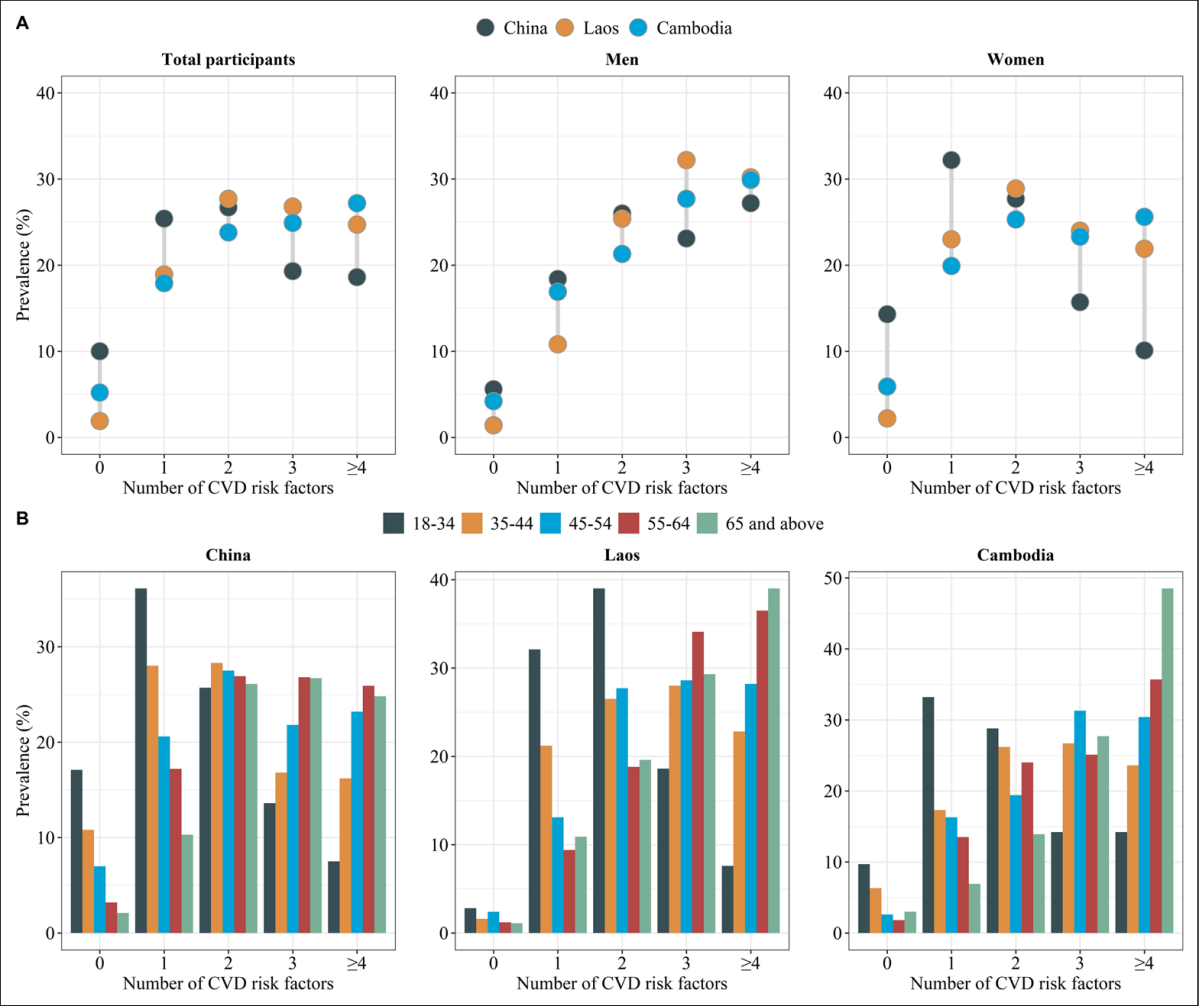
The cardiovascular disease clustering of risk factors in sex or age groups in China, Laos, and Cambodia.

Prevalence without CVD risk factors was 10% in China, 1.9% in Laos, and 5.2% in Cambodia. In the three regions, 64.6%, 79.2%, and 76.0% had ≥2 CVD risk factors, respectively; 37.9%, 51.5%, and 52.1% had ≥3 CVD risk factors, respectively. In the male participants, the proportion of individuals without risk factors was notably low across all three regions, with percentages of 5.6%, 1.4%, and 4.2% for China, Laos, and Cambodia, respectively. We also observed that Chinese men predominantly exhibited two risk factors (25.8%), Lao men were more likely to have a combination of three risk factors (32.2%), and four or more (29.9%) were more likely in Cambodian men. Among women, the prevalence of 0, 1, and 2 risk factors was higher in all three countries compared to men, while the prevalence of 3, 4, and more risk factors was lower than in men. Chinese women primarily exhibited a single risk factor, whereas Laos and Cambodia predominantly displayed 2 and ≥4 risk factors, respectively (Figure 3A).

Among participants aged 18–34 years in all three countries, the prevalence of individuals without risk factors was most pronounced. Notably, China exhibited a higher percentage (17.1%) in this age group, compared to Laos (2.8%) and Cambodia (9.7%). More women tended to possess only one CVD risk factor (Supplementary Table S3). In China, participants in the 18–34 age group predominantly exhibited one risk factor (36.1%), while the 35–64 age group showed a higher tendency for a combination of two risk factors (28.3%). Participants aged 65 and above mainly demonstrated three risk factors. Across all three countries, the proportion of individuals with 0 and 1 risk factors exhibited a clear decreasing trend with increasing age groups, while the proportion of those with clustered four or more risk factors increased as the age group advanced, all *p* < 0.001 (Figure 3B). For the CVD risk factor distribution, details have been presented in Supplementary Table S4.

### Multivariate logistic regression analysis of CVD risk factor clustering

As shown in Table 2, the odds ratio (OR) for the aggregation of ≥2 modifiable CVD risk factors was significantly higher in men than women, especially in China (China: OR, 3.35; 95% *CI*, 3.03–3.70, *p <* 0.001; Laos: OR, 2.22; 95% *CI*, 1.53–3.21, *p <* 0.001; Cambodia: OR, 1.39 95% *CI*, 1.02–1.90, *p <* 0.05), including ≥3 and ≥4 followed the same trend. Comparing the 18–34 age group as the reference, the propensity for clustering ≥2, ≥3, and ≥4 risk factors substantially increased across the advancing age groups in all three countries. In contrast to the main ethnic groups, ethnic minorities in China and Cambodia displayed a higher rate of clustering ≥2 and ≥3 risk factors. Higher levels of education in the Chinese population were associated with a lower of risk factor aggregation. However, in Cambodia, the risk of aggregating ≥2 risk factors was elevated among individuals with primary and post-primary education. Participants with low educational attainment in Laos were also associated with a risk of having three or more CVD risk factors.

**Table 2:** Multivariable logistic analysis of the clustering of cardiovascular disease risk factors in China, Laos, and Cambodia.


	NUMBER OF CARDIOVASCULAR DISEASE RISK FACTORS, OR (95% *CI*)								

	≥2			≥3			≥4		
		
CHARACTERISTIC	CHINA	LAOS	CAMBODIA	CHINA	LAOS	CAMBODIA	CHINA	LAOS	CAMBODIA

**Sex**									

**Women**	1 [Reference]	1 [Reference]	1 [Reference]	1 [Reference]	1 [Reference]	1 [Reference]	1 [Reference]	1 [Reference]	1 [Reference]

**Men**	3.35 (3.03–3.70)^b^	2.22 (1.53–3.21)^b^	1.39 (1.02–1.90)^a^	3.68 (3.33–4.08)^c^	1.71 (1.28–2.28)^c^	1.42 (1.10–1.84)^a^	4.01 (3.52–4.56)^b^	1.36 (1.00–1.84)^a^	1.34 (1.01–1.80)^a^

**Age group**									

**18–34**	1 [Reference]	1 [Reference]	1 [Reference]	1 [Reference]	1 [Reference]	1 [Reference]	1 [Reference]	1 [Reference]	1 [Reference]

**35–44**	1.79 (1.58–2.03)^b^	1.83 (1.20–2.79)^a^	2.52 (1.73–3.66)^b^	1.82 (1.58–2.09)^b^	2.89 (1.93–4.31)^b^	2.57 (1.80–3.68)^b^	2.31 (1.91–2.79)^b^	3.59 (2.07–6.24)^b^	1.84 (1.18–2.88)^a^

**45–54**	2.87 (2.48–3.32)^b^	2.89 (1.84–4.54)^b^	3.09 (1.97–4.86)^b^	2.94 (2.54–3.40)^b^	3.88 (2.56–5.87)^b^	4.06 (2.72–0.06)^b^	3.48 (2.88–4.21)^b^	4.74 (2.79–8.06)^b^	2.63 (1.64–4.21)^b^

**55–64**	4.13 (3.44–4.95)^b^	4.32 (2.49–7.47)^b^	4.25 (2.51–7.20)^b^	3.93 (3.33–4.63)^b^	6.82 (4.35–10.70)^b^	3.90 (2.54–5.98)^b^	3.91 (3.18–4.81)^b^	6.82 (3.99–11.66)^b^	3.30 (2.02–5.39)^b^

**≥65**	6.23 (5.09–7.63)^b^	3.57 (2.14–5.96)^b^	7.43 (3.56–15.51)^b^	4.82 (4.08–5.70)^b^	6.03 (3.87–9.41)^b^	7.59 (4.38–13.15)^b^	5.29 (4.32–6.47)^b^	7.48 (4.41–12.69)^b^	5.30 (3.08–9.15)^b^

**Ethnic group**									

**Main ethnic group**	1 [Reference]	1 [Reference]	1 [Reference]	1 [Reference]	1 [Reference]	1 [Reference]	1 [Reference]	1 [Reference]	1 [Reference]

**Ethnic group**	1.14 (1.03–1.27)^a^	0.92 (0.50–1.68)	2.56 (1.84–3.56)^b^	1.16 (1.05–1.29)^a^	0.63 (0.39–1.03)	1.92 (1.51–2.50)^b^	1.03 (0.91–1.18)	0.76 (0.47–1.24)	1.75 (1.33–2.31)^b^

**Education**									

**No education**	1 [Reference]	1 [Reference]	1 [Reference]	1 [Reference]	1 [Reference]	1 [Reference]	1 [Reference]	1 [Reference]	1 [Reference]

**Primary school**	0.60 (0.48–0.74)^b^	1.21 (0.82–1.79)	1.67 (1.16–2.39)^a^	0.67 (0.56–0.79)^b^	1.45 (1.05–2.00)^a^	1.08 (0.80–1.45)	0.74 (0.61–0.90)^b^	1.18 (0.83–1.68)	0.98 (0.71–1.35)

**Junior high school and above**	0.45 (0.36–0.76)^b^	1.16 (0.75–1.80)	1.61 (1.05–2.47)^a^	0.48 (0.40–0.57)^b^	1.30 (0.89–1.90)	1.14 (0.78–1.67)	0.54 (0.44–0.66)^b^	1.19 (0.77–1.85)	0.83 (0.54–1.29)


Abbreviation: ^a^Indicates *P* < 0.05, ^b^Indicates *P* < 0.001.

## Discussion

This study adopted the same survey scheme through China, Laos, and Cambodia, which border each other, and all are located in the Lancang-Mekong River region. To our knowledge, this is the first population-based cross-country study concerning the epidemiology of CVD risk factors in the less developed Lancang-Mekong River area. The results of the epidemiological characteristics, clustering, and influencing factors of eight modifiable CVD risk factors in the three countries showed: (1) the primary metabolic risk factor in Laos and Cambodia was hypertension, whereas it was overweight/obesity in China; (2) insufficient consumption of vegetables and fruits and insufficient physical activity were the two biggest behavioral risk variables in all three regions; and (3) propensity for CVD risk factors clustering was higher in men and increased with age in all three regions.

In comparison with the previous survey [18], our findings showed that the prevalence of hypertension, DM, and dyslipidemia was higher in all three regions along the Lancang-Mekong River. Among the metabolic risk factors, the prevalence of hypertension is the most common. However, Cambodia (23.49%) had the lowest prevalence of hypertension among the three regions, consistent with previous studies from 10 countries in South-East Asia (13.89%) [18]. Given that hypertension independently heightens the risk of multiple CVDs (acute myocardial infarction and stroke), particularly in the elderly [30]. However, in developing countries, hypertension is usually not timely detected and treated [31,32]. China has updated guidelines for managing hypertension [33], diabetes [34], and dyslipidemia [35], whereas Laos and Cambodia lack such comprehensive guidelines. It’s recommended that these countries consider adopting international guidelines such as the European Society of Hypertension and European Society of Cardiology [36], aligning with their own contexts and healthcare systems, to establish effective CVD prevention and risk factors control measures. In this cross-sectional study, we found that the prevalence of overweight/obesity was ranked as the top CVD metabolic risk factor in Yunnan province, China, our finding was similar to previous studies [37,38]. A survey in 2020 also showed that China’s overweight and obesity rate is higher than 50% [39], and the prevention and control of CVD should emphasize this critical trend. Furthermore, we observed that the most prevalent clustering of the two and three risk factor combinations was that of “hypertension + overweight/obesity” or “hypertension + dyslipidemia + overweight/obesity”, these findings were consistent with the findings from Nanjing city, China. It suggests that following China, Laos, and Cambodia expect changes in economic lifestyle and diet, especially with the low accessibility of health resources in low-income areas, overweight/obesity will become another major metabolic risk factor for the residents along the Lancang-Mekong River Basin in the future.

Previous studies [18,40] have also found a significantly higher proportion of inadequate vegetable and fruit intake in China, Laos, and Cambodia. Globally, inadequate vegetable and fruit consumption has contributed to almost 3 million annual heart disease and stroke-related deaths [41]. Moreover, a meta-analysis highlighted the inverse correlation between increased vegetable and fruit consumption and cardiovascular mortality [42]. In the Lancang-Mekong countries with good accessibility to vegetable and fruit, public health authorities should prioritize education and initiatives promoting their intake, as this could be a cost-effective approach for CVD prevention. Additionally, physical inactivity was alarmingly high, especially in Cambodia. A study also found that more than a quarter of adults globally did not get enough physical activity in 2016 [43]. In 2018, the WHO launched Global action plan on physical activity 2018– 2030 [44] and set a target to reduce physical inactivity by 15% by 2030. Overall, unhealthy lifestyles remain prevalent in all three regions, which highlights the trend in LMICs and further underscores the importance of addressing physical inactivity through awareness campaigns and interventions, in line with the WHO Global Action Plan.

The main finding of this study was the higher prevalence of CVD clustering than in previous studies. For example, a study from six provinces in China [13] showed that 43.3% of the population had ≥2 CVD risk factors, and 16.7% had 3 or more. Another study [45] reported that the prevalence of ≥2 and ≥3 CVD risk factors in adult was 33%, and 14%, respectively. When compared with the findings from Guangxi province of China, 17.9%, 36.9%, and 45.3% of participants had zero, one, and at least two CVD risk factors [46]. Our study revealed a high prevalence of CVD risk factors clustering across all three LMICs. The highest occurrence of four or more risk factors was observed in Cambodia [27.2%], while the lowest was in China (18.6%). CVD risk factors cluster are more serious in the Lancang-Mekong River region of this study. It has been shown that the combination of risk factors multiplies the incidence of cardiovascular disease [12]. Early detection of certain clusters of risk variables can help identify high-risk individuals and enable tailored preventive efforts to reduce the occurrence of CVD and adverse outcomes. It is worth mentioning that the proportion of no-risk factor aggregation is the highest in China and the lowest in Laos among the three regions. The potential reason for this could be that the Chinese government has started a new healthcare reform program as early as 2009, proposing a package of basic public health services [47]. Primary care physician-led public health services is essential for reducing the burden of blood pressure and blood glucose, improving health literacy, and reducing the burden of CVD risk factors, especially in Yunnan province, China [19]. Therefore, we suggest that public health departments in the Lancang-Mekong Basin should consider this potential health strategy.

At the same time, our research revealed that men in three regions were more likely to have a cluster of CVD risk factors (containing ≥2, ≥3, and ≥4). Several other studies have similarly found gender differences in CVD risk factor clustering [13,15,45]. The distribution of CVD risk factors depicted in our study, after stratification by sex, demonstrated a similar trend, with higher rates of smoking and alcohol consumption in men than in women. The prevalence of CVD risk factors clustering increased with age, in agreement with other previous studies [37]. For nations facing a significant cardiometabolic burden due to unprecedented population aging, it is crucial to identify the distinct risk factors profiles of various age groups in order to promote personalized management strategies to lower CVD risk and mortality [4]. Therefore, men and the elderly should be the main targets of early preventive initiatives that screen for the cluster of CVD risk factors.

This study shows that there are similarities and differences in CVD risk factors across the three regions, and therefore there is an urgent need for proactive strategies to prevent CVD in the Lancang-Mekong region. Recommendations include adopting tailored guidelines, addressing emerging risk factors like obesity, and promoting healthier lifestyles. The high prevalence of risk factors clustering emphasizes the importance of early detection and targeted interventions, especially among men and the elderly.

## Limitations

Our study has the following limitations. First, the small sample sizes from Oudomxay province in Laos and Ratanakiri province in Cambodia due to low population density, as well as the fact that this study was conducted during the COVID-19 pandemic, which also limited the collection of samples, coupled with the diverse sampling methods, could limit the generalizability of these findings. Furthermore, limited data on treatment and control of hypertension, diabetes, and dyslipidemia among these participants could have affected the study’s comprehensive assessment of CVD risk factors and burdens in LMICs.

Despite these limitations, Rigorous measures were taken to maintain consistency in data collection through standardized data collection procedures, uniform questionnaires and certified instruments used by trained enumerators in all survey centers, thus avoiding measurement bias in the results. In addition, the same variable definitions and disease diagnostic criteria were used in the statistical analyses, improving comparability between samples.

## Conclusions

Our study found that the prevalence of CVD risk factors and aggregation was high among adults in the low- and middle-income areas along Lancang-Mekong River Basins. It is recommended that cross-regional cooperation be implemented to target multiple CVD risk factors and implement tailored public health prevention approaches, such as the development of targeted CVD and chronic disease prevention guidelines to effectively tackle the burden of CVD in LMICs.

## Data Accessibility Statement

The data supporting the findings of this study and its supplementary material are available on request from the corresponding author.

## Additional Files

The additional files for this article can be found as follows:

10.5334/gh.1319.s1 Supplementary Table S1. Detrended Oscillation and Clock Parameters.

10.5334/gh.1319.s2 Supplementary Table S2. Prevalence and cluster of behavioral risk factors in China, Laos, and Cambodia.

10.5334/gh.1319.s3 Supplementary Table S3. Demographic characteristics of participants without CVD risk factor clusters in China, Laos, and Cambodia.

10.5334/gh.1319.s4 Supplementary Table S4. Demographic characteristics of participants with CVD risk factors aggregation in China, Laos, and Cambodia.

10.5334/gh.1319.s5 Supplementary Figure S1. Three survey provinces from China Laos and Cambodia long Lancang-Mekong River.
