# Supplementary material for: Prevalence and Clustering of Cardiovascular Disease Risk Factors among Adults Along the Lancang-Mekong River: A Cross-Sectional Study from Low- and Middle-Income Countries
Source: Glob Heart. 2024 Apr 17;19(1):35. doi: 10.5334/gh.1319 (PMC11025572; doi:10.5334/gh.1319)
Supplement: Supplementary Table S1. — Detrended Oscillation and Clock Parameters. [file gh-19-1-1319-s1.pdf]

Manuscript

Supplementary Material

1 Supplementary Figures and Tables

1.1 Supplementary Figures

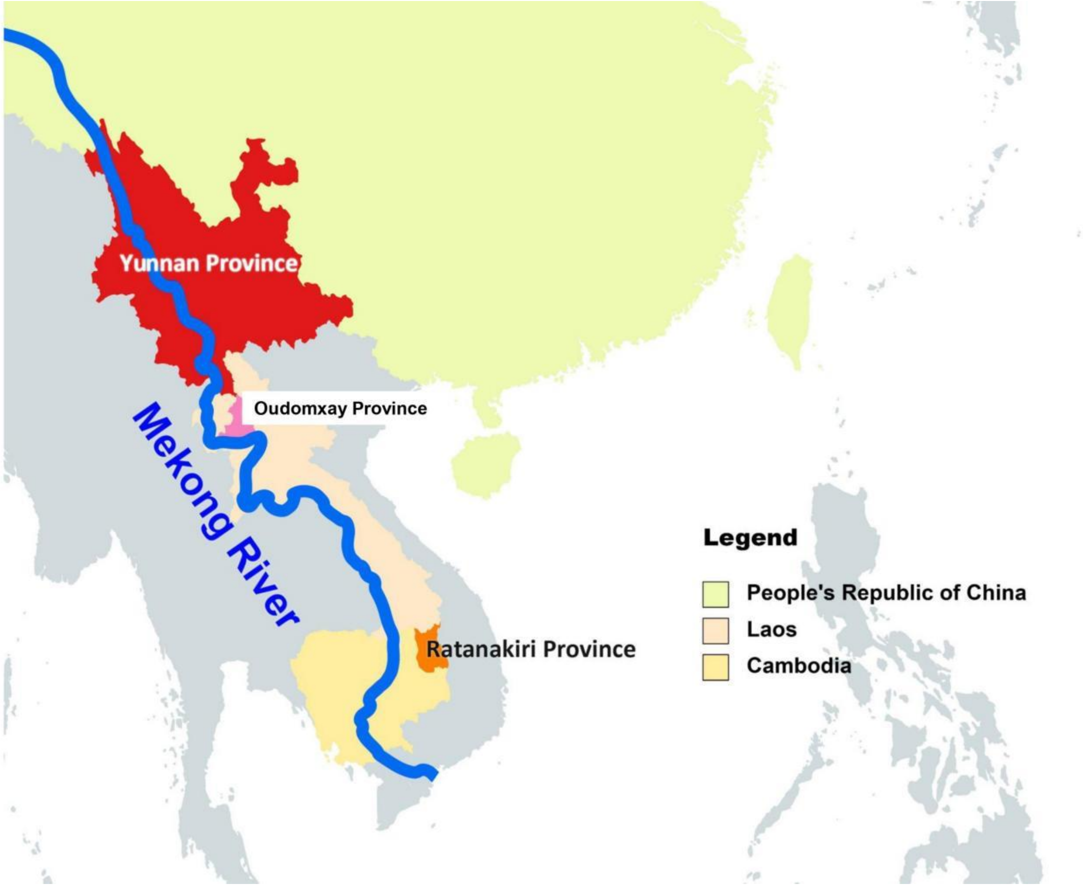

Supplementary Figure 1. Three survey provinces from China Laos and Cambodia long Lancang-Mekong River

1.2 Supplementary Figures

Supplementary Table S1. Prevalence and cluster of metabolic risk factors in China, Laos, and Cambodia

	**Total (n=11005)**	**China (n=8859)**	**Laos (n=1039)**	**Cambodia (n=1107)**
**a**	3616 (32.9)	2974 (33.6)	382 (36.8)	260 (23.5)
**a+b**	1445 (13.1)	1291 (14.6)	82 (7.9)	72 (6.5)
**a+c**	663 (6.0)	407 (4.6)	177 (17.0)	79 (7.1)

**a+d**	1635 (14.9)	1481 (16.7)	106 (10.2)	48 (4.3)
**a+b+c**	254 (2.3)	192 (2.2)	42 (4.0)	20 (1.8)
**a+b+d**	744 (6.8)	694 (7.8)	30 (2.9)	20 (1.8)
**a+c+d**	299 (2.7)	233 (2.6)	46 (4.4)	20 (1.8)
**a+b+c+d**	140 (1.3)	120 (1.4)	14 (1.3)	6 (0.5)
**b**	3159 (28.7)	2810 (31.7)	154 (14.8)	195 (17.6)
**b+c**	393 (2.6)	278 (3.1)	73 (7.0)	42 (3.8)
**b+d**	1296 (11.8)	1203 (13.6)	45 (4.3)	48 (4.3)
**b+c+d**	198 (1.8)	157 (1.8)	26 (2.5)	15 (1.4)
**c**	1209 (11.0)	617 (7.1)	339 (32.6)	253 (22.9)
**c+d**	461 (4.2)	325 (3.7)	81 (7.8)	55 (5.0)
**d**	3467 (31.5)	3068 (34.6)	219 (21.1)	180 (16.3)

Abbreviation: + Indicatesco-existence of risk factors; hypertension=a， hypercholesterolemia=b， DM=c， overweight and obesity=d

Supplementary Table S2. Prevalence and cluster of behavioral risk factors in China, Laos, and Cambodia

	**Total (n=11005)**	**China (n=8859)**	**Laos (n=1039)**	**Cambodia (n=1107)**
**a**	2143(19.5)	1588 (17.9)	158 (15.2)	397 (35.9)
**a+b**	824 (7.5)	570 (6.4)	67 (6.4)	187 (16.9)
**a+c**	723 (6.6)	351 (4.0)	39 (3.8)	333 (30.1)
**a+d**	1365 (12.4)	1013 (11.4)	149 (14.3)	203 (18.3)
**a+b+c**	316 (2.9)	137 (1.5)	7 (0.7)	172 (15.5)
**a+b+d**	404 (3.7)	264 (3.0)	63 (6.1)	77 (7.0)

**a+c+d**	396 (3.6)	204 (2.3)	37 (3.6)	155 (14.0)
**a+b+c+d**	159 (1.4)	80 (0.9)	7 (0.7)	72 (6.5)
**b**	2022 (18.4)	1420 (16.0)	254 (24.4)	348 (31.4)
**b+c**	634 (5.8)	321 (3.6)	46 (4.4)	267 (24.1)
**b+d**	1226 (11.1)	818 (9.2)	234 (22.5)	174 (15.7)
**b+c+d**	324 (2.9)	159 (1.8)	41 (3.9)	124 (11.2)
**c**	3244 (29.5)	2297 (25.9)	304 (29.3)	643 (58.1)
**c+d**	1665 (15.1)	1059 (12.0)	272 (26.2)	334 (30.2)
**d**	6244 (56.7)	4659 (52.6)	936 (90.1)	646 (58.4)

Abbreviation: + Indicatesco-existence of risk factors; smoker= a， drinker= b， Insufficient physical activity= c， Insufficient intake of vegetable and fruit =d

Supplementary Table S3. Demographic characteristics of participants without cardiovascular disease risk factor clusters in China, Laos, and Cambodia

	**the number of CVD risk factors**							
	**0 (n=965)**				**1 (n=2650)**			
	**China (n=887)**	**Laos (n=20)**	**Cambodia (n=58)**	***P* value**	**China (n=2246)**	**Laos (n=196)**	**Cambodia (n=208)**	***P* value**
**Sex**								
**Men**	247 (27.8)	5 (25.0)	17 (29.3)	0.931	805 (35.8)	38 (19.4)	69 (33.2)	<0.001
**Women**	640 (72.2)	15 (75.0)	41 (70.7)		1441 (64.2)	158 (80.6)	139 (66.8)	
**Age, y**								
**18-34**	529 (59.6)	8 (40.0)	22 (37.9)	0.02	1118 (49.8)	93 (47.4)	75 (36.1)	0.001
**35-44**	187 (21.1)	3 (15.0)	24 (41.4)		486 (21.6)	40 (20.4)	66 (31.7)	
**45-54**	107 (12.1)	5 (4.2)	6 (10.3）		315 (14.0)	27 (13.8)	37 (17.8)	
**55-64**	33 (3.7)	2 (5.3)	3 (5.2）		176 (7.8)	16 (8.2)	23 (11.1)	
**≥65**	31 (3.5)	2 (10.0)	3 (5.2)		151 (6.7)	20 (10.2)	7 (3.4)	
**ethnic**								
**Main ethnic group**	610 (68.8)	3 (15.0)	30 (51.7)	<0.001	1566 (69.7)	12 (6.1)	137 (65.9)	<0.001

**Ethnic group**	277 (31.2)	17 (85.0)	28 (48.3)		680 (30.3)	184 (93.9)	71 (34.1)	
**Education**								
**No education**	28 (3.2)	8 (40.0)	32 (55.2)	<0.001	123 (5.5)	59 (30.1)	76 (36.5)	<0.001
**Primary school**	96 (10.8)	4 (20.0)	16 (27.6)		378 (16.8)	70 (35.7)	68 (32.7)	
**Junior high school and above**	763 (86.0)	4 (20.0)	10 (17.2)		1745 (77.7)	67 (34.2)	64 (30.8)	

Supplementary Table S4. Demographic characteristics of participants with cardiovascular disease risk factors aggregation in China, Laos, and Cambodia

	the number of CVD risk factors											
	2 (n=2920)				3 (n=2268)				≥4 (n=2202)			
	China (n=2368)	Laos (n=288)	Cambodia (n=264)	P value	China (n=1714)	Laos (n=278)	Cambodia (n=276)	P value	China (n=1644)	Laos (n=257)	Cambodia (n=301)	P value
**Sex**												
**Men**	1129 (47.7)	89 (30.9)	87 (33.0)	<0.001	1012 (59.0)	113 (40.6)	113 (40.9)	<0.001	1190 (72.4)	106 (41.2)	122 (40.5)	<0.001
**Women**	1239 (52.3)	199 (69.1)	177 (67.0)		702 (41.0)	165 (59.4)	163 (59.1)		454 (27.6)	151 (58.8)	179 (59.5)	
**Age, y**												
**18-34**	795 (33.6)	113 (39.2)	65 (24.6)	<0.001	420 (24.5)	54 (19.4)	32 (11.6)	<0.001	232 (14.1)	22 (8.6)	32 (10.6)	<0.001

**35-44**	492 (20.8)	50 (17.4)	100 (37.9)		292 (17.0)	53 (19.1)	102 (37.0)		281 (17.1)	43 (16.7)	90 (29.9)	
**45-54**	421 (17.8)	57 (19.8)	44 (16.7)		334 (19.5)	59 (21.2)	71 (25.7)		355 (21.6)	58 (22.6)	69 (22.9)	
**55-64**	276 (11.7)	32 (11.1)	41 (15.5）		275 (16.0)	58 (20.9)	43 (15.6)		265 (16.1)	62 (24.1)	61 (20.3)	
**≥65**	384 (16.2)	36 (12.5)	14 (5.3)		393 (22.9)	54 (19.4)	28 (10.1)		511 (31.1)	72 (28.0)	49 (16.3)	
**ethnic**												
**Main ethnic group**	1666 (70.4)	15 (5.2)	139 (52.7)	<0.001	1171 (68.3)	30 (10.8)	121 (43.8)	<0.001	1177 (71.6)	32 (12.5)	114 (37.9)	<0.001
**Ethnic group**	702 (29.6)	273 (94.8)	125 (47.3)		543 (31.7)	248 (89.2)	155 (56.2)		467 (28.4)	225 (87.5)	187 (62.1)	
**Education**												
**No education**	266 (15.5)	84 (30.2)	122 (44.2)	<0.001	269 (16.4)	88 (34.2)	157 (52.2)	<0.001	266 (15.5)	84 (30.2)	122 (44.2)	<0.001
**Primary school**	445 (26.0)	123 (44.2)	97 (35.1)		521 (31.7)	113 (44.0)	102 (33.9)		445 (26.0)	123 (44.2)	97 (35.1)	
**Junior high school and above**	1003 (58.5)	71 (25.5)	57 (20.7)		854 (51.9)	56 (21.8)	42 (14.0)		1003 (58.5)	71 (25.5)	57 (20.7)	
